# Effect of Perioperative Immunonutrition on Early-Postoperative Complications in Patients Undergoing Radical Cystectomy for Bladder Cancer: A Case Series

**DOI:** 10.3390/jcm14061992

**Published:** 2025-03-15

**Authors:** Francesco Cianflone, Alice Tartara, Lucia Aretano, Valentina Da Prat, Andrea Ringressi, Carlo Marchetti, Chiara Lonati, Giulia Gambini, Riccardo Caccialanza, Richard Naspro

**Affiliations:** 1 Department of Urology, Fondazione IRCCS Policlinico San Matteo, 27100 Pavia, Italy; 2 Clinical Nutrition and Dietetics Unit, Fondazione IRCCS Policlinico San Matteo, 27100 Pavia, Italy; 3 Department of Urology, Spedali Civili of Brescia, 25123 Brescia, Italy; 4 Clinical Epidemiology and Biometry Service, Fondazione IRCCS Policlinico San Matteo, 27100 Pavia, Italy

**Keywords:** immunonutrition, cystectomy, bladder cancer, complications

## Abstract

**Objective:** The objective was to evaluate the impact of perioperative immunonutrition (IN) on postoperative complications in patients undergoing radical cystectomy (RC) for bladder cancer (BC). **Methods:** A prospective case series of 19 patients treated with perioperative IN between October 2022 and July 2023 was conducted. Patients received preoperative IN based on nutritional risk and postoperative IN with gradual recovery of normal feeding. The inclusion criteria encompassed clinically node-negative patients without metastatic disease. The outcomes were assessed using Clavien–Dindo classification and included infectious complications, wound healing disorders, ileus, anemia, genitourinary issues, recovery time, and compliance with the nutritional regimen. **Results:** Sixteen patients (84.2%) experienced complications. Most were low-grade (CD 1–2), with no CD > 3a. Wound disorders affected 10.5% and anemia requiring transfusion occurred in 47.4% of patients, infectious complications were reported in 26.3%, and ileus in 36.8%. The median time to first flatus was 2 days (IQR 2–3), while resumption of oral feeding occurred after 4 days (IQR 2–5), like mobilization (IQR 2–5). The median hospital stay was 14 days (IQR 11–18). Compliance with IN was 78.9%, with gastrointestinal intolerance being the primary cause of discontinuation. **Conclusions:** Patients with RC undergoing perioperative IN showed low rates of high-grade complications and promising results in bowel function recovery and infection rates. Further randomized controlled trials are required to validate these results.

## 1. Introduction

Radical cystectomy (RC) with pelvic lymph node dissection (PLND) and urinary diversion is the treatment of choice in T2–T4a N0M0 muscle-invasive bladder cancer and in very-high-risk non-muscle-invasive bladder cancer (NMIBC), bacillus of Calmette-Guérin (BCG)-refractory, BCG-relapsing, and BCG-unresponsive NMIBC [1,2].

However, this procedure is still burdened by an elevated rate of perioperative complications, not only when performed with a laparotomic approach, but also when carried out in a minimally invasive manner [3,4].

One of the emerging factors that might play a role in patients’ recovery after this surgery is their preoperative nutritional status: the prevalence of malnutrition in the RC population is estimated to range from 21.3% to 55% using the Nutritional Risk Screening (NRS)-2002 tool, and patients at risk of malnutrition are more prone to post-surgical complications after major urological surgery [5,6].

Moreover, nutritional status before RC, defined by the assessment of preoperative weight loss, body mass index, and serum albumin, is a strong predictor of poor overall survival and 90-day mortality [7,8,9]. In fact, the perioperative period is characterized by an inflammatory and hypermetabolic state that leads to a loss of muscle mass [10]. Muscle mass plays a critical role in maintaining body homeostasis and metabolism [10]. During periods of high energy demand or low energy supply, amino acids stored as protein in muscle tissue are broken down to provide energy through gluconeogenesis [10]. Moreover, amino acid metabolism is significantly affected by stress-related responses [11].

Previous studies have shown that arginine levels decrease after surgery, and low levels of this essential amino acid may inhibit T-cell function, increasing susceptibility to infection, wound breakdown, and further muscle loss post surgery [12]. Similarly, glutamine, a non-essential amino acid crucial for the functioning of the immune system, also decreases under stress conditions [13]. Therefore, maintenance and restoration of muscle mass with optimal nutritional strategies are crucially important [14].

In other settings, mainly gastrointestinal surgery, different randomized controlled trials (RCTs) have been published assessing the beneficial clinical impact of oral nutritional support, especially immunonutrition (IN), in terms of postoperative complications and length of stay (LOS) [15].

The term “immunonutrition” refers to the potential effect of specific nutrients, such as glutamine, arginine, n-3 fatty acids (FAs), and RNA, on the immune system, as they can enhance the host’s defense mechanisms in response to catabolic stress.

On the contrary, little is known about the impact of IN on patients with RC due to the small sample size of the available studies [16,17]. To this end, our report aimed to shed some light on this issue.

## 2. Methods

### 2.1. Patient Cohort

Between October 2022 and July 2023, a consecutive prospective group of patients receiving preoperative and postoperative IN was enrolled for this study.

Patients who were clinically node-positive and/or who had metastatic disease upon preoperative staging were excluded.

Furthermore, due to the composition of the oral nutritional supplements (ONSs) used, specific exclusion criteria were considered to select patients eligible for immunonutrition:

- Inability to understand the correct way of taking ONSs (e.g., language barrier, absence of a home caregiver);
- Diabetes mellitus type 1;
- Non-compensated diabetes mellitus type 2 (glycated hemoglobin > 7% or fasting blood glucose level > 130 mg/dL);
- Inability to take ONS as a consequence of the presence of pre-existing disease such as dysphagia.

All patients were preoperatively assessed by the anesthesiologist according current European guidelines for elective noncardiac surgery [18].

In the immunonutrition group, patients were assessed by nutritionists pre admission and received nutritional counseling and a personalized diet plan based on their nutritional needs, estimated using the 24 h dietary recall method.

The immunonutrition protocol included drinking an ONS enriched with immunonutrients (Impact Oral^®^, Nestlé Health Science, Vevey, Switzerland, containing arginine, ω-3 fatty acids, and nucleotides; 334 kcal per dose) 2 times a day for 5 or 7 days before surgery, according to the nutritional risk of the patient (5 days for a patient not at risk and 7 days for a patient at nutritional risk). After surgery, when the patient could start eating again, the immunonutritional protocol included the following:

- A complex-carbohydrate-based liquid ONS (Preop^®^, Danone Nutricia S.p.A., Milan, Italy maltodextrin, 100 kcal, protein 0 g, lipid 0 g) given 2 times a day on the first day;
- Preop^®^ 2 times a day + a high-protein, high-calorie liquid ONS (Fortimel Compact Protein^®^, 306 kcal, protein 18 g) on the second day;
- 2 Preop^®^ + 2 Fortimel Compact Protein on the third day;
- Gradual introduction of an oral diet from the fourth day onwards, with transition to a free diet on the seventh day. If the patient was still unable to start oral feeding 24–48 h after surgery, a nutritional assessment was required.

During this assessment, the nutritionist, based on laboratory exams and the patient’s nutritional risk, decided whether to initiate parenteral nutrition (PN) or continue with intravenous therapy, with daily reassessment for the initiation of the refeeding protocol. In this study, there were no specific criteria for the timing of PN prescription, which was left to the discretion of the physician, taking into account the patient’s baseline nutritional status and the predicted duration of fasting.

The criteria used to assess the possibility of resuming eating included the absence of nausea, the presence of intestinal gas passage, and the absence of an acute infectious condition.

### 2.2. Outcomes

The primary outcome was reporting on the rates of early-postoperative complications (defined as those occurring within 30 days of surgery), including infectious complications, wound healing disorders, postoperative ileus, anemia, and genitourinary complications classified according to the Clavien–Dindo (CD) classification. Genitourinary complications were defined as evidence of renal failure, ureteral anastomosis leak, ureteral anastomosis stenosis, and hydronephrosis; wound disorders included superficial wound dehiscence, fascial dehiscence, and complete surgical wound dehiscence; finally, infectious complications comprised urinary tract infection, sepsis, abdominopelvic abscess, pyelonephritis, pneumonia, and wound infection.

The secondary endpoints were the following:

- To report on the time to recovery of bowel function, time to postoperative oral feeding, time to mobilization, rate of use of PN, and length of hospital stay;
- To assess the compliance rate with the use of perioperative immuno-ONS, evaluating the reasons why patients discontinued the intake of nutritional supplements.

### 2.3. Covariates

Relevant demographic and tumor-related data were recorded. The demographic data included patient age (at the time of surgery), gender, ASA (American Society of Anesthesiologists) score, Charlson comorbidity index (CCI), and NRS-2002 [19]. Intraoperative data, including intraoperative complications, were recorded.

### 2.4. Statistical Analysis

Descriptive statistics were expressed as the median and interquartile range (IQR) or the number with percentage for continuous and non-numeric variables, respectively.

Statistical analyses were performed using STATA14.0 (Stata Corp., College Station, TX, USA).

## 3. Results

Data from 19 consecutive patients undergoing IN were prospectively accrued out of 23 who underwent RC for organ-confined BC in our institution at the same time. Of the four patients excluded, three patients were due to non-compensated diabetes mellitus and one due to their inability to understand the correct way of taking ONSs, caused by a language barrier. Most of the patients were male, with a median age of 71 years (67–82). The median BMI was 25.1 kg/m^2^ (IQR 23.3–27.9), while the median CCI was 3 (IQR 2–3). All patients underwent radical cystectomy for clinical organ-confined disease, with only one patient having a cT > 2, and only one patient underwent neoadjuvant chemotherapy. Eleven (57.9%) patients had a previous abdominal surgical intervention, and over 80% of patients had an ASA score of 2. The median NSR-2002 was 2 (IQR 0–3). No patient underwent preoperative bowel preparation, and the Early Recovery After Surgery (ERAS) protocol was not employed (Table 1).

Thirteen patients (68.4%) underwent an ureteroileocutaneostomy as urinary diversion, five (26.3%) underwent an ureterocutaneostomy, and only one patient underwent a neobladder reconstruction (5.3%). In addition, seven patients (36.8%) underwent a robot-assisted approach and fifteen (78.9%) pelvic lymphadenectomy. Considering the final pathological staging on histological examination, three patients (15.7%) had pT4a disease, one (5.3%) had pT3b, five (26.3%) had pT3a, four (21.1%) had pT2, and five (26.3%) had pT1, while one patient (5.3%) had a bladder free from disease. Moreover, concomitant CIS was found in three (15.7%) patients. In addition, 15.8% of the population showed pN+ disease (Table 2).

Overall, sixteen patients (84.2%) experienced complications of any grade, with a median number of complications per patient of 2 (IQR 1–2). The majority of the more severe complications per patient were of a low grade: 52.5% of the patients (n = 10) experienced CD grade 2 complications, while 21% were CD 1 (n = 4). Moreover, while no genitourinary complications were recorded, wound disorders affected 10.5% (n = 2) of the patients, and anemia requiring transfusions affected 47.4% (n = 9) of the cohort. Only two patients (12.5%) experienced a CD grade 3a complication, whilst no higher-grade complications were recorded. In addition, 36.8% (n = 7) of the patients experienced ileus, while 26.3% (n = 5) developed infectious complications. Twelve patients (63.2%) required a gastric tube, with a median duration of gastric tube maintenance of 3.5 days (IQR 2.5–3.5). Parenteral nutrition was used in three patients (15.8%) with a median duration of 12 days (IQR 8–18). The median time to the first flatus was 2 days (IQR 2–3), and the median resumption of oral nutrition was 4 days (IQR 2–5). Moreover, the median time to mobilization was 4 days (IQR 2–5), and the median length of hospital stay was 14 days (IQR 11–18).

Lastly, the compliance rate to immunonutrition was 78.9%: four patients discontinued the preoperative immunonutritional supplements due to the onset of gastrointestinal intolerance in the first 48 h, experiencing a sense of swollen stomach, nausea, abdominal distension, and diarrhea. In every one of these patients, IN was promptly discontinued, and gastrointestinal distress was resolved within two days of its onset (Table 3).

## 4. Discussion

Recently, special emphasis has been given to the nutritional status of surgical patients. Preoperative malnutrition and underfeeding have been recognized as risk factors for higher complication rates after RC, poorer survival outcomes, and higher healthcare costs [6,8]. ESPEN guidelines from the European Society for Clinical Nutrition and Metabolism recommend assessing the nutritional status before and after major surgery, in order to identify and support patients with malnutrition and those at nutritional risk before surgery, and maintain nutritional status in the postoperative period, when severe catabolism and prolonged fasting can occur [20]. Many authors have focused on enhancing nutritional status by combining standard ONS with so-called “immunonutrients” (e.g., arginine, dietary nucleotides, ω-3 fatty acids). These immunonutrients exert an immunomodulatory effect and improve protein synthesis after surgery [21]. Several studies have demonstrated that perioperative immunonutrition is associated with a reduction in infectious complications and a shorter length of stay in patients undergoing major gastrointestinal surgery [21,22,23]. Nevertheless, there are limited data on the effect of immunonutrition in patients undergoing RC for BC. Recently, a narrative review described the state of the art of the available scientific literature on the topic of immunonutrition in RC for BC [24]. In our cohort, the complication rate was a little higher compared to the literature [4,20]. These results might have been due to different factors, including age (elderly patients with a median age of 72 years vs. 70 and 68 years in previous studies) and comorbidities (median CCI 2.5 [IQR 2–3], and ASA score > 2 in 28% of patients). Moreover, the rate of low-grade complications higher than previously reported might also be attributable to the thorough prospective data collection that was employed in this study.

In fact, in a systematic review and meta-analysis of randomized controlled trials with meta-regression analyses, Katsimperis et al. reported that the most common complications were gastrointestinal complications (20%), infectious complications (17%), and ileus (14%), with the majority of complications occurring being Clavien I-II (45%) [25]. These rates showed some complications having rates lower than the ones we reported; however, as was pointed out in a reply by Montorsi et al., the reported morbidity might not reflect the outcomes of procedures performed in the community [26].

In a prospective RCT from 2016, Hamilton-Reeves et al. did not find any differences in terms of the overall complication rate within 30 days of surgery when comparing patients undergoing RC who received an ONS containing arginine to patients who received a nutritional supplement without arginine [27]. On the contrary, in 2014, Bertrand J. et al. found that IN reduced the complication rate by 37% [28]. Nevertheless, we interestingly observed that the patients in our cohort had a very low rate of high-grade complications, with none higher than CD 3a. Similarly, in Bertrand’s 2014 study, the Clavien grades for complications were lower in patients receiving IN [28]. Moreover, many studies have shown that IN reduces the rate of postoperative infectious complications and postoperative ileus [27,28,29]. However, one study by Cozzi et al. in 2012 failed to demonstrate significant differences in terms of postoperative ileus and wound disorders in patients receiving IN [30]. In our series, the patients in the IN group did not experience genitourinary complications, but certainly the effect of IN on reducing anastomotic leakage needs to be evaluated by comparative and possibly prospectively randomized studies. In addition, seven patients underwent a robotic procedure, which might have acted as a confounder. However, we hypothesize that the surgical approach may have only had a moderate effect on these outcomes, as several trials have actually found no advantage for robot-assisted techniques over standard open surgery for patients undergoing RC in terms of complication rate (except for blood loss requiring transfusions) and hospital stay [31,32,33]. Moreover, two recent meta-analyses reported as the main driver of complication the choice of urinary diversion, with comparable effects between the robot-assisted and open approaches [34,35].

Similarly to Patel et al., who reported an improvement in restoration of bowel function, in our cohort, we found a short time to the first flatus (median 2, IQR 2–3) [36]. In line with previous studies [27,36], which showed a compliance rate ranging from 71% to 100%, 78.9% (15/19) of our patients took all the immunonutritional oral supplements.

It is noteworthy that, although neoadjuvant chemotherapy (NAC) is the standard of care for localized MIBC, in our department, NAC has unfortunately not been available for all patients as recommended due to various organizational and patient eligibility-related reasons. It is known that NAC may contribute to skeletal muscle wasting in patients with cancer and that common adverse effects, such as reduced appetite and early satiety sensation, may affect food intake and body weight. One study demonstrated that NAC-related nutritional deterioration represents an independent risk factor for any postoperative complication, in particular ileus and infections [31]. In our series, only one patient received NAC, but this did not lead to worse outcomes (LOS = 11 days, without postoperative complications). Therefore, the effect of NAC on nutritional status should be considered in future studies, according to the authors.

In addition, the optimal immunonutrient supplementation regimen for patients with RC has not yet been identified, nor the ideal target population. In our series, all patients were assessed by nutritionists pre admission and received nutritional counseling, a personalized dietary plan based on their nutritional requirements, and preoperative IN based on their nutritional risk (NRS-2002).

In some studies, immunonutritional supplementation was recommended only during the preoperative period, while in others it continued after RC, with promising outcomes and a good compliance rate. The duration of supplementation was various in previous studies, ranging from 5 to 7 days before surgery and from 5 to 14 days after surgery [27]. The type and the dosage also varied from study to study, with some regimens requiring one carton per day, other three per day, and some four per day, with the compliance rate not being influenced by this factor [27]. The nutritional risk was not reported in all previous studies; nevertheless, the results were promising regardless of the nutritional risk [27]. In our study, we decided to provide perioperative IN supplements to all patients, differentiating between a five-day and a seven-day preoperative regimen based on each patient’s nutritional risk, as assessed by nutritionists during the pre-admission evaluation. This approach aimed to tailor IN to the specific needs of each patient and bridge the nutritional gap before surgery, for both those who were at nutritional risk and those who were not at the time of preoperative evaluation.

Our study has several limits, mainly due to the small sample size and the lack of a control group. In addition, its inclusion of both robot-assisted and traditional approaches, alongside its single-center nature, could have acted as an additional confounder. These factors, when combined, indeed limit the generalizability of our findings. However, while assessing the impact of robotic approaches would have been paramount given their potential of acting as a confounder, it was not feasible to proceed with a comparison between the two surgical approaches in our study due to the restricted sample size of the overall cohort and, particularly, robotic cases (n = 7). In addition, it could be argued the study’s strict exclusion criteria might have introduce selection bias by excluding all patients with non-compensated diabetes, given that patients with diabetes are generally more susceptible to postoperative complications [37]. Furthermore, we did not calculate the global costs, but cost-effectiveness studies are required in the future, taking into account total parenteral nutrition consumption and length of hospital stay, as well as therapies and interventions required to resolve each complication.

However, our limited experience can provide some insight into the postoperative outcomes after a complex surgical procedure such as RC in patients receiving IN, supporting its feasibility and safety.

One of the strengths of our report is the strict inclusion criteria and the thoroughly designed IN protocol, which might provide a solid basis for further in-depth research.

In conclusion, high-quality trials with more rigorous standardization of intervention protocols are needed. Ideally, these should be multicentric and randomized, with a sufficiently large sample size to enable comprehensive analyses and address potential strong confounders, such as the choice of surgical approach. Such studies are essential to provide greater clarity in this poorly investigated field.

## 5. Conclusions

We present our initial experience in the use of IN in patients undergoing RC and their perioperative outcomes. However, well-designed randomized control trials are needed to collect more consistent, comparable, and uniform data without biases. These trials will be essential to assess the effectiveness of IN and determine the most appropriate dosage, timing, and duration of this kind of support.

## Figures and Tables

**Table 1:** Demographic and clinical characteristics.

**Number of patients**	19
**Age, median (IQR)**	71 (67–82)
**Gender**	
**Male, *n* (%)**	14 (73.7%)
**Female, *n* (%)**	5 (26.3%)
**BMI, median (IQR)**	25.1 (23.3–27.9)
**Age-adjusted CCI, median (IQR)**	6 (4–6)
**CCI, median (IQR)**	3 (2–3)
**Clinical tumor staging, *n* (%)**	
T1	2 (10.5%)
T2	16 (84.2%)
T3	1 (5.3%)
**Clinical nodal staging, *n* (%)**	
N0	19 (100%)
N1, N2, N3	0 (0%)
**Neoadjuvant chemotherapy, *n* (%)**	1 (5.3%)
**Smoking habits, *n* (%)**	
Nonsmoker	8 (42.1%)
Active smoker	5 (26.3%)
Previous smoker	6 (31.6%)
**ASA score, *n* (%)**	
ASA 2	16 (84.2%)
ASA 3	2 (10.5%)
ASA 4	1 (5.3%)
**Prior abdominal surgery, *n* (%)**	11 (57.9%)
**NRS-2002, *n* (%)**	2 (0–3)
**NLR, median (IQR)**	3.22 (2.08–4.25)

BMI: body mass index; CCI: Charlson comorbidity index; age-adjusted CCI: adjusted Charlson comorbidity index according to age; ASA: American Society of Anesthesiologists; NRS-2002: Nutritional Risk Screening; and NLR: neutrophil-to-lymphocyte ratio.

**Table 2:** Intraoperative and pathological characteristics.

**Urinary Diversion, n (%)**	
Ureterocutaneostomy	5 (26.3%)
Ureteroileocutaneostomy	13 (68.4%)
Neobladder	1 (5.3%)
**Robotic surgical approach, n (%)**	7 (36.8%)
**Lymph node dissection, n (%)**	15 (78.9%)
**Pathological tumor stage, n (%)**	
T0	1 (5.3%)
T1	5 (26.3%)
T2	4 (21.1%)
T3a	5 (26.3%)
T3b	1 (5.3%)
T4a	3 (15.7%)
**Pathological nodal stage, n (%)**	
N0	12 (63.2%)
N1	1 (5.3%)
N2	0
N3	2 (10.5%)
Nx	4 (21.1%)
**Concomitant CIS, n (%)**	3 (15.7%)
**Concomitant HV, n (%)**	2 (10.5%)
**Concomitant LVI, n (%)**	15 (78.9%)

HV: histological variant; LVI: lymphovascular invasion.

**Table 3:** Postoperative complication.

**More Severe Clavien–Dindo Grade, n (%)**	
Clavien–Dindo 1	4 (25%)
Clavien–Dindo 2	10 (62.5%)
Clavien–Dindo 3a	2 (12.5%)
Clavien–Dindo 3b, 4a, 4b, and 5	15 (78.9%)
**Genitourinary complications, n (%)**	0 (0%)
**Wound disorders, n (%)**	2 (10.5%)
**Anemia, n (%)**	9 (47.4%)
**Infectious complications, n (%)**	5 (26.3%)
**Ileus, n (%)**	7 (36.8%)

Genitourinary complications: renal failure, ureteral anastomosis leak, ureteral anastomosis stenosis, and hydronephrosis; wound disorders: superficial wound dehiscence, fascial dehiscence, and complete surgical wound dehiscence; and infectious complications: urinary tract infections, sepsis, abdominopelvic abscess, pyelonephritis, pneumonia, and wound infection.

## Data Availability

The original contributions presented in this study are included in the article. Further inquiries can be directed to the corresponding author.
